# Fungus-Mediated Green Synthesis of Silver Nanoparticles Using *Aspergillus terreus*

**DOI:** 10.3390/ijms13010466

**Published:** 2011-12-29

**Authors:** Guangquan Li, Dan He, Yongqing Qian, Buyuan Guan, Song Gao, Yan Cui, Koji Yokoyama, Li Wang

**Affiliations:** 1 Department of Pathogenobiology, Norman Bethune College of Medicine, Jilin University Mycology Research Center, Jilin University, Changchun 130021, China; E-Mails: guangquanli83@gmail.com (G.L.); hedan@jlu.edu.cn (D.H.); qianyq09@mails.jlu.edu.cn (Y.Q.); songgao850118@gmail.com (S.G.); cuiyan923@hotmail.com (Y.C.); 2 State Key Laboratory of Inorganic Synthesis and Preparative Chemistry, College of Chemistry, Jilin University, Changchun 130012, China; E-Mail: guanbuyuan@gmail.com; 3 Medical Mycology Research Center, Chiba University, Chiba 260-8673, Japan; E-Mail: yoko@faculty.chiba-u.jp

**Keywords:** silver nanoparticles, *Aspergillus terreus*, biosynthesis, NADH

## Abstract

The biosynthesis of nanoparticles has received increasing attention due to the growing need to develop safe, cost-effective and environmentally friendly technologies for nano-materials synthesis. In this report, silver nanoparticles (AgNPs) were synthesized using a reduction of aqueous Ag^+^ ion with the culture supernatants of *Aspergillus terreus*. The reaction occurred at ambient temperature and in a few hours. The bioreduction of AgNPs was monitored by ultraviolet-visible spectroscopy, and the AgNPs obtained were characterized by transmission electron microscopy and X-ray diffraction. The synthesized AgNPs were polydispersed spherical particles ranging in size from 1 to 20 nm and stabilized in the solution. Reduced nicotinamide adenine dinucleotide (NADH) was found to be an important reducing agent for the biosynthesis, and the formation of AgNPs might be an enzyme-mediated extracellular reaction process. Furthermore, the antimicrobial potential of AgNPs was systematically evaluated. The synthesized AgNPs could efficiently inhibit various pathogenic organisms, including bacteria and fungi. The current research opens a new avenue for the green synthesis of nano-materials.

## 1. Introduction

Nanoparticles (NP) are usually clusters of atoms in the size range of 1–100 nm. It is understood that the properties of a metal NP are determined by its size, shape, composition, crystallinity, and structure [1]. As an important metal, silver nanoparticles (AgNPs) have a number of applications, from electronics [2] and catalysis [3] to infection prevention [4] and medical diagnosis [5]. For example, AgNPs could be used as substrates for Surface Enhanced Raman Scattering (SERS) to probe single molecules [6], and also useful catalysts for the oxidation of methanol to formaldehyde [7]. AgNPs has been known as excellent antimicrobial and anti-inflammatory agents, and thus were used to improve wound healing [8]. To date, a number of physical and chemical strategies were employed for the synthesis of AgNPs [9,10]. However, concern has been raised on the toxicity of chemical agents used in AgNPs synthesis. Thus, it is essential to develop a green approach for AgNPs production without using hazardous substances to the human health and environment.

Compared with the traditional synthetic methods, biological systems provide a novel idea for the production of nano-materials [11]. Up to now, several microorganisms from bacteria to fungi have been reported to synthesize inorganic materials either intra- or extracellularly, and thus to be potentially utilized as eco-friendly nanofactories [12,13]. *Pseudomonas stutzeri* AG259, isolated from silver mines, has been shown to produce silver nanoparticles [14], and the bioreduction of Ag was also reported in *Bacillus licheniformis.* Recently a further advancement in the biological synthesis approach was shown by demonstrating that the shape of Ag nanoparticles could be tuned from nanospheres to nanoprisms by controlling the growth kinetics of a silver resistant bacteria *Morganella psychrotolerans* [15]. Moreover, the same research group also demonstrated that all the members of the genus *Morganella* were capable of synthesizing extracellular Ag nanoparticles, which was correlated to silver resistance machinery operating in these organisms [16] Compared with bacteria, fungi have been known to secrete much higher amounts of bioactive substances, which made fungi more suitable for large-scale production [17]. In addition, the extracellular biosynthesis using fungi could also make downstream processing much easier than bacteria [13]. An interesting example of the biosynthesis using fungi was that the cell-associated biosynthesis of silver using *Fusarium oxysporum* was demonstrated by Ahmad *et al.*, and the particles were overall quasi-spherical with size range between 5 and 15 nm [18]. There also have been several reports on the biosynthesis of AgNPs using fungi, including *Fusarium acuminatum* [19] and *Penicillium fellutanum* [20]. Despite these impressive results, the origins of fungi having the ability for AgNPs synthesis were still limited, and the detailed mechanism was still not well elucidated. Previous reports have shown that a large number of active substances secreted by fungi played important roles as reducing agents and capping agents in the reaction [21]. Therefore, it was of great significance to explore novel fungi strain for synthesizing AgNPs based on the biodiversity. More importantly, it could also facilitate the deeper understanding of molecular mechanism for AgNPs biosynthesis.

Herein, we investigated the biosynthesis of AgNPs using *Aspergillus terreus* and its underlying mechanism. The properties of obtained AgNPs were characterized by ultraviolet-visible spectroscopy, transmission electron microscopy (TEM) and X-ray diffraction (XRD) techniques. Furthermore, the key factors controlling the reaction and the antimicrobial activity of AgNPs synthesized were evaluated. This work provided a potential for the production of AgNPs without the involvement of toxic chemicals and radiation.

## 2. Results and Discussion

### 2.1. Synthesis and Characterization of AgNPs Using *Aspergillus terreus*

In this study, AgNPs were synthesized using a reduction of aqueous Ag^+^ with the culture supernatants of *Aspergillus terreus* at room temperature.

**Figure 1:**
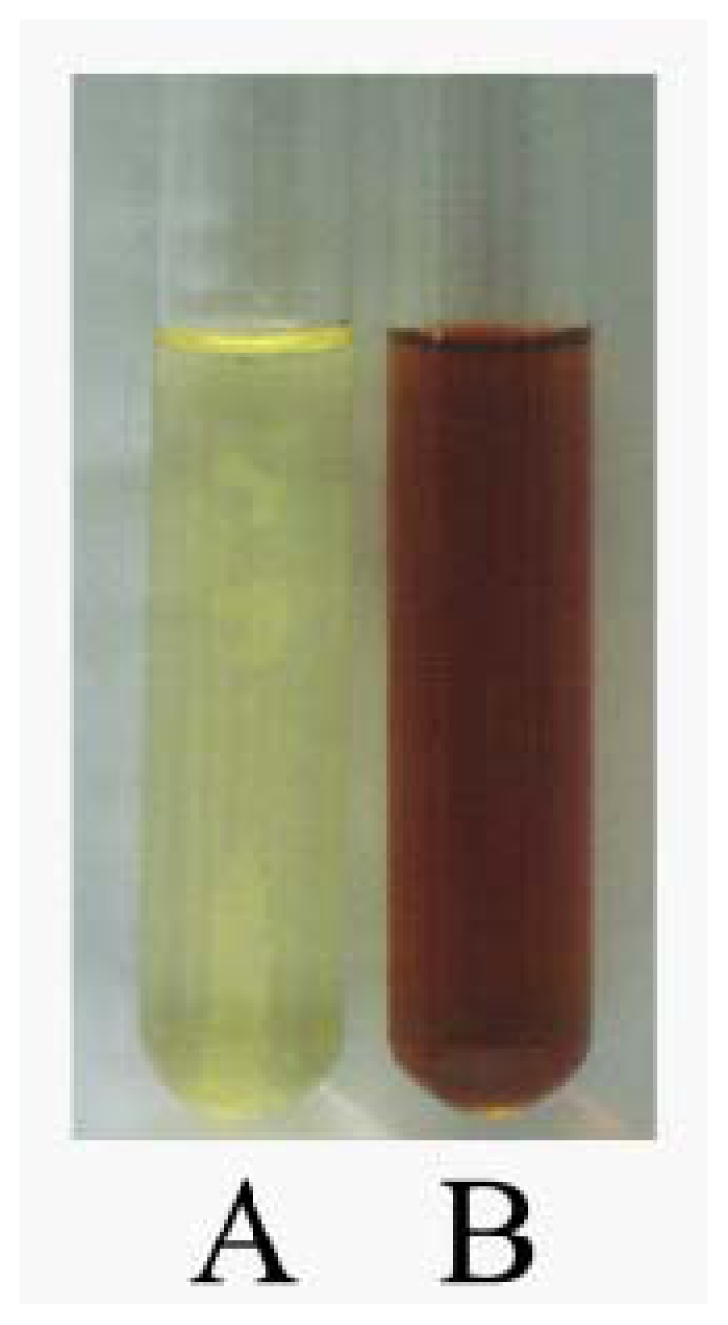
The crude cell filtrate of *Aspergillus terreus* mixed without AgNO_3_ (**A**) and with AgNO_3_ (**B**) after 24 h.

It was generally recognized that AgNPs produced brown solution in water, due to the surface plasmon resonances (SPR) effect and reduction of AgNO_3_ [22]. After the addition of AgNO_3_ solution, the crude cell filtrate of *A. terreus* changed from light yellow to brown in a few hours, while no color change was observed in the culture supernatant without AgNO_3_ (Figure 1). Thus, color change of the solution clearly indicated the formation of AgNPs. The color intensity of the cell filtrate with AgNO_3_ was sustained even after 24 h incubation, which indicated that the particles were well dispersed in the solution, and there was no obvious aggregation.

**Figure 2:**
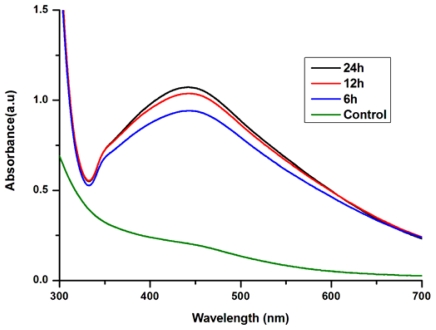
The UV-Vis spectra recorded for the reaction of fungal cell filtrate with AgNO_3_ solution.

All these reactions were monitored by ultraviolet-visible spectroscopy of the colloidal AgNPs solutions. The ultraviolet-visible spectra of the cell filtrate with AgNO_3_ showed a strong broad peak at 440 nm (SPR band), which indicated the presence of AgNPs (Figure 2). These results were consistent with the reports of Naik *et al.* and Verma *et al.* [23,24]. The intensity of the SPR band steadily increased from 6 h to 24 h as a function of time of reaction. It was also observed that the AgNPs formed were quite stable in the supernatant of *A. terreus*.

**Figure 3:**
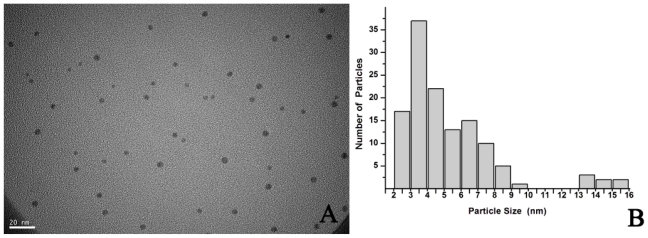
(**A**) Representative images of AgNPs synthesized by the reduction of AgNO_3_ solution with the crude cell filtrate from *Aspergillus terreus*; (**B**) Size distribution of the AgNPs from TEM analysis.

**Figure 4:**
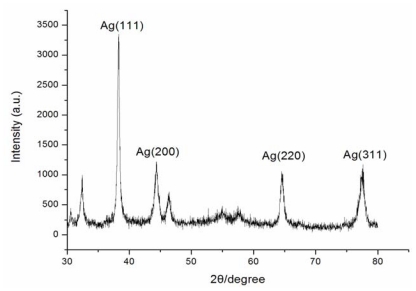
Representative X-ray diffraction patterns of AgNPs synthesized by *Aspergillus terreus* (a.u. = arbitrary units).

The application of AgNPs was highly dependent on the chemical composition, shape, size, and monodispersity of particles [25]. To broaden the application scope, the AgNPs obtained were systematically characterized using TEM and XRD analysis. Through the TEM analysis, the particles were spherical and polydisperse with an average size of 4.3 nm (1–20 nm), and the majority of the particles were less than 10 nm (Figure 3). For the crystalline nature of the AgNPs, intense XRD peaks were observed corresponding to the (111), (200), (220), (311) planes at 2θ angles of 38.28°, 44.38°, 64.54°, and 77.64°, respectively (Figure 4). This was in good agreement with the unit cell of the face centered cubic (fcc) structure (JCPDS File No. 04-0783) with a lattice parameter of a = 4.077 Å. Some intense diffraction peaks at 2θ angles of 32.05°, 46.05°, 54.6° and 57.3°, might be related to AgCl which was owing to the chloride ions involved during preparation of the cell filtrate. Because of the biomass residue, other crystallographic impurities were also observed in the XRD profile. The size of AgNPs according to the XRD was about 5.2 nm. This result was consistent with the TEM study.

### 2.2. Key Factors Governing the Biosynthesis of AgNPs

**Figure 5:**
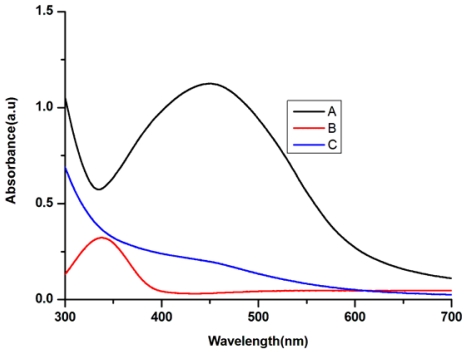
The UV-Vis spectra recorded for the reaction of dialyzed fungal cell filtrate with AgNO_3_ solution. Curve A corresponds to the dialyzed cell filtrate with NADH and AgNO_3_ solution; Curve B corresponds to NADH alone with AgNO_3_ solution; Curve C corresponds to the dialyzed cell filtrate with AgNO_3_ solution.

In the present study, the key factors in the supernatant of *A. terreus* governing the formation of AgNPs were investigated. The AgNO_3_ solution was added to the dialyzed cell filtrate under the same conditions for the synthesis of AgNPs. However, the color of reaction mixture did not change over 24 h. Meanwhile, the absence of SPR at 440 nm for the reaction mixture of dialyzed cell filtrate (Figure 5C) indicated that the reaction was highly dependent on an active substance with a low molecular weight (<7000 Da). Interestingly, when NADH was added to the dialyzed cell filtrate, the reaction was recovered in a few minutes, and the band at 440 nm was almost as strong as that in the crude cell filtrate (Figure 5A). These results indicated that NADH might be a key factor for the synthesis of AgNPs by *A. terreus*. As a negative control, when NADH alone was added to the AgNO_3_ solution, no band was observed at 440 nm (Figure 5B), which confirmed that NADH alone was not sufficient for the reaction to occur, and other active substances with higher molecular weight might also play important roles in the reduction of silver ions.

**Figure 6:**
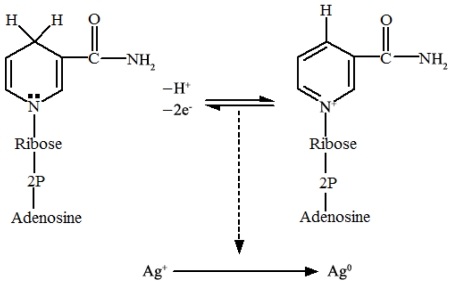
Schematic representation of the biosynthesis of AgNPs related to NADH, NADH-dependent reductase was also essential for the reaction.

In organism, NADH is a widespread reduced coenzyme involved in redox reaction, and can be used as a reducing agent by many enzymes *in vivo* [26,27]. Thus, we interfered that a NADH-dependent reductase released by *A. terreus* might account for the synthesis of AgNPs. In the process, NADH acted as an electron carrier, and the silver ions obtained electrons from NADH via the NADH-dependent reductase, and then were reduced to Ag (Figure 6). Similarly, there were other reports that NADH-dependent reductases were believed to be involved in the biosynthesis of AgNPs in *F. oxysporum* and *P. fellutanum* [20,28,29]. These findings might be of great significance to the development of a continuous biological production of nanoparticles at a large scale through the addition of NADH or couple with the regenerating system of co-enzymes.

**Figure 7:**
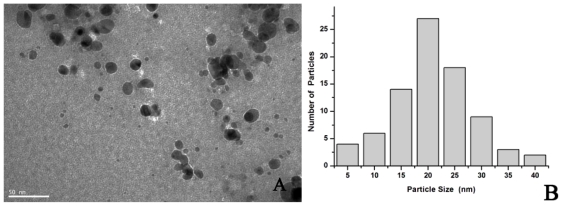
(**A**) Representative images of AgNPs synthesized by the reduction of AgNO_3_ solution with the dialyzed cell filtrate and NADH; (**B**) Size distribution of the AgNPs from TEM analysis.

Representative TEM images of AgNPs film which was derived from dialyzed cell filtrate with the addition of NADH were shown in Figure 7. The AgNPs were spherical or nearly spherical, which was same as the ones synthesized by crude cell filtrate. Nevertheless, the size of nanospheres was about 21.3 nm on average and in the range of 10–30 nm, a little larger than the ones synthesized by crude cell filtrate. This size difference might be caused by different concentrations of NADH in these two reaction mixtures, and the presence or absence of other small molecules (might as stabilizers). Therefore, the concentration of reducing agent and the presence of stabilizer were crucial to the size and shape control of nanoparticles in such a biosynthesis system.

### 2.3. Antimicrobial Activity Analysis of AgNPs

The antimicrobial activity of AgNPs against various pathogenic organisms including bacteria and fungi was investigated. Compared with the control, the diameters of inhibition zones increased for all the test pathogens (Table 1). The AgNPs produced could inhibit three different typical pathogenic bacteria, including *Staphylococcus aureu*, *Pseudomonas aeruginosa* and *Escherichia coli*, as previously described [23,30]. Thus, AgNPs could be considered as excellent broad-spectrum antibacterial agents. More importantly, the AgNPs produced by *A. terreus* exhibited potent antifungal activity against *Candida* species, which were the most important pathogenic fungi. Additionally, the AgNPs showed good inhibition activity towards two kinds of filamentous fungus, which were naturally resistant to the common antifungal agent Fluconazole [31]. Since the biosynthesized AgNPs showed considerable antifungal activity, they could be potential to be widely used in clinical applications.

## 3. Experimental Section

### 3.1. Materials

*A. terreus* (BC0603) was isolated from soil, and maintained on potato dextrose agar (PDA) medium at 28 °C. The isolated fungus was identified using morphological characteristics and mitochondrial cytochrome *b* gene analysis [32]. Three kinds of bacteria were tested for their susceptibility for AgNPs: *S. aureu*, *E. coli* and *P. aeruginosa*. Six kinds of fungi were all tested for its antifungal effect: *C. albicans*, *C. parapsilosis*, *C. krusei*, *C. tropicalis*, *A. fumigatus* and *A. flavus. A. terreus* and all strains used in the study were stored in Jilin University Mycology Research Center (JUMRC).

Two medium (potato dextrose agar and potato dextrose broth) were purchased from BD (Becton, Dickinson and company Co., Sparks, MD, USA). The chemical silver nitrate (AgNO_3_) and NADH were purchased from Sigma-Aldrich (St. Louis, MO, USA), and used as received.

### 3.2. Biomass Preparation

To prepare biomass for biosynthesis, *A. terreus* was grown in potato dextrose broth liquid medium (PDB). The flasks were inoculated with spores and incubated at 28 °C on a rotary shaker (120 rpm) for 96 h. The biomass was harvested by filtration through filter paper (Whatman filter paper No. 1), and then washed with distilled water to remove any components of the medium. 25 g biomass (wet weight) was placed in individual flasks containing 100 mL Milli-Q water. The flasks were incubated under the conditions described above for 24 h. The biomass was filtered, and the crude cell filtrate was collected for subsequent experiment.

### 3.3. Biosynthesis of AgNPs

AgNPs were synthesized using 50 mL cell filtrate mixed with 10 mL AgNO_3_ solution (10 mmol/L) in a 250 mL Erlenmeyer flask incubated at 28 °C in dark for 24 h. A flask with no addition of silver ion was used as control. AgNPs were concentrated by centrifugation of the reaction mixture at 10,000 rpm for 10 min twice, and then were collected for further characterization.

### 3.4. Characterization of AgNPs

The bioreduction of Ag^+^ in aqueous solution was monitored using an ultraviolet-visible spectrophotometer (Shimadzu UV-2550) from 240 to 750 nm, at a resolution of 1 nm.

The dried reaction mixture embedded with AgNPs was used for XRD analysis. XRD patterns were recorded on RINT2000 vertical goniometer operated at a voltage of 50 kV and current of 200 mA with Cu Kα radiation (λ = 1.5405 Å), and the diffracted intensities were recorded from 30° to 80° 2θ angles.

For TEM analysis, a drop of aqueous solution containing AgNPs were placed on the carbon coated copper grids and dried by allowing water to evaporate at room temperature. Micrographs were obtained using a Tecnai F20 S-Twin (USA) operating at 200 kV. The size of AgNPs were estimated from the Debye-Scherrer Eq by determine the width of the (111) Bragg reflection [2,33], and size distribution of the resulting nanoparticles was also estimated on the basis of TEM micrographs.

### 3.5. Investigation of the Key Factors of Reaction

To investigate the key factors of reaction, some of crude cell filtrate was dialyzed (molecular weight cut-off = 7 kDa) against distilled water for 48 h at 4 °C to remove small molecular weight compounds. 200 μL NADH (20 mmol/L) was then added to the dialyzed cell filtrate. Afterwards, AgNO_3_ solution (10 mmol/L) was added under the same conditions as above. Control reactions were performed without the addition of cell filtrate or NADH.

### 3.6. The Antimicrobial Activity Analysis of AgNPs

The antimicrobial activity of AgNPs synthesized from *A. terreus* against *P. aeruginosa*, *S. aureus*, *E. coli*, *C. albicans*, *C. krusei*, *C. glabrata*, *C. tropicalis*, *A. fumigatus* and *A. flavus* was investigated using a disk diffusion assay. The disk diffusion assay was carried out using the Oxford cup method [34]. Each strain was swabbed uniformly onto individual plates, and a concentrated solution of AgNPs was poured into each cup (20 μg per cup) on all the plates. After incubation at 37 °C or 28 °C for 24 h, the diameter of inhibition zone was measured using caliper. AgNO_3_ (10 mmol/L) was used individually as the negative control. The assays were performed in triplicate.

## 4. Conclusions

In this study, AgNPs were synthesized extracellularly by *A. terreus* at room temperature. The AgNPs were quite stable without using any toxic chemicals as capping agents. The spherical AgNPs ranged in size from 1 to 20 nm, and showed promising broad-spectrum antimicrobial activity. NADH and NADH-dependent reductase were probably the key factors for the biosynthesis of AgNPs. The ability to synthesize AgNPs as potential anti-microbial agents using *A. terreus* is highly promising for the green, sustainable production of nano-metals, and also enhances its widespread application as an important strategy.

## Figures and Tables

**Table 1:** Size of the inhibition zone for AgNPs synthesized by *Aspergillus terreus* against the tested microorganisms.

Tested Pathogenic Organisms	Mean Size of Inhibition Zone (mm)	
	Control	Test
*Candida albicans* (ATCC 90028)	9	16 ± 1
*Candida krusei* (ATCC 6258)	10	14 ± 2
*Candida parapsilosis* (ATCC 22019)	9	13 ± 1
*Candida tropicalis* (JLCC 30394)	10	14 ± 1
*Aspergillus flavus* (IFM 55648)	9	13 ± 2
*Aspergillus fumigates* (IFM 40808)	9	14 ± 2
*Staphylococcus aureus* (ATCC 25923)	9	16 ± 1
*Pseudomonas aeruginosa* (ATCC 27853)	9	12 ± 1
*Escherichia coli* (ATCC 25922)	10	13 ± 1

ATCC: American Type Culture Collection, USA; IFM: Institute for Food Microbiology (at present the Medical Mycology Research Center, Chiba University), Japan; JLCC: Culture Collection of Jilin University, Mycology Research Center, China; Control: AgNO_3_; Test: AgNPs.
